# The well-being of Iranian adult citizens; is it related to mental health literacy?

**DOI:** 10.3389/fpsyt.2023.1127639

**Published:** 2023-05-05

**Authors:** Seyed Mohammad Hossein Mahmoodi, Maryam Rasoulian, Elaheh Khodadoust, Zahra Jabari, Sahar Emami, Masoud Ahmadzad-Asl

**Affiliations:** ^1^Mental Health Research Center, School of Behavioral Sciences and Mental Health, Iran University of Medical Sciences, Tehran, Iran; ^2^Faculty of Medicine, Iran University of Medical Sciences, Tehran, Iran; ^3^Mental Health Research Center, Tehran Institute of Psychiatry, Iran University of Medical Sciences, Tehran, Iran

**Keywords:** subjective well-being, mental health literacy, mental health, positive mental health, depression screening

## Abstract

**Background:**

Subjective well-being (SWB) is a fundamental concept in the definition of mental health and is a significant health indicator for individuals and societies. Mental health literacy (MHL) is a modifiable variable with known effects on mental health, but its relationship with SWB is not recognized. In this study, the SWB is measured, and its relationship to MHL is investigated.

**Methods:**

In this cross-sectional study conducted in Iran in 2019, 1,682 individuals participated using a convenient sampling method. Participants with a basic ability to use internet were included. A simple online form was used to collect data. SWB and MHL were measured with three questionnaires: WHO-5 Well-Being Index, Mental Health Literacy Scale, and Mental Health Positive Knowledge.

**Results:**

Most of the participants were young (mean age 25.99, SD 9.14), female (71.9%), and had a university degree (78.5%). The mean SWB was 50.19 out of 100 (SD 20.92). More than half of the participants (50.4%) were screen-positive for clinical depression regarding their low well-being. Significant but very small correlations were detected between SWB and both MHL measures.

**Conclusion:**

The well-being of half of the educated Iranian citizens who participated in this study was poor and lower than previous measurements. No strong correlation is detected between SWB and MHL measures in this study. This suggests that people’s well-being cannot be improved by merely implementing mental health educational programs.

## 1. Introduction

The concept of well-being is fundamental in the definition of mental health and is considered an outcome variable of the person’s whole life (1, 2). It is also proposed as an indicator of human development in communities and is therefore crucial at both individual and societal levels (2, 3). Although difficult to define, well-being is considered an individual’s beliefs and feelings about the extent their life is going well (4). Therefore, it is a subjective evaluation that can be cognitive or affective and is recommended to be assessed as a subjective measure (4, 5). The affective part is more related to the hedonic perspective of well-being, and the cognitive aspects are more associated with the eudaimonic view; these are the two basic paradigms that construct the meaning of subjective well-being (SWB) for individuals (6, 7).

There has been an enormous growth in SWB studies in the last two decades (4). According to current empirical findings, higher SWB is related to many positive outcomes, including health and help-seeking behavior, longevity, social relationships, citizenship and organizational success, productivity, and resilience (4, 5, 8, 9). On the other hand, higher SWB results from external objective factors like better health, higher income, stronger social relationship, and more religiosity, along with internal mental factors such as personality, comparison processes, needs, and desires (4).

Worldwide inequality in SWB has been rising in recent years, and middle-east is among the regions with more inequality and the lowest reported well-being (2, 10). Several studies estimating Iranian’s SWB are available with variable results. In the recent World Happiness Report, Iran ranked low, 118th among 153 nations (11). Similarly, Iranians showed relatively low SWB in many other national and international investigations (12–14). In a national survey of Iranian young adults’ happiness in 2017, however, better results were reported (15).

Mental health literacy (MHL), also a growing study field, is a modifiable factor that influences mental health in various ways (16–18). It is recently formulated as a four-domain construct: (1) understanding how to maintain and improve mental health, which is known as positive MHL, (2) understanding mental disorders and treatments, (3) mental illness stigma, and (4) competency for help-seeking behavior (19). Higher MHL improves help-seeking, treatment compliance, relationship with health staff, attitude toward mental health issues, and lessens stigma (16, 17).

As MHL is one of the predictors of mental health, and mental health is, in turn, one of the determinants of SWB, the relationship between MHL and SWB is tested in a few studies. No association between SWB and MHL is detected in recent investigations of Australian adolescents, British university students, and sports coaches (8, 9, 20). On the other hand, a significant small correlation was reported in a study of SWB and positive MHL in Norwegian youths (21). In Iran, a study detected a small correlation between the quality of life of the general population of a city and MHL, while another recent study found no correlation between the general health of female adolescents and MHL (22, 23). Such findings may have practical implications as MHL is a modifiable factor that can be improved through education (18, 24).

There are inconsistent reports about Iranian citizens’ well-being. Besides, the country has experienced rapid economic changes in recent years. Therefore, it is necessary to have up-to-date SWB estimations. To improve SWB, a candidate may be promoting MHL as it is modifiable by the health system, but current evidence is not conclusive about such a relationship, and studies had mixed results. Therefore, in this study, we aimed to (1) estimate SWB in a sample of Iranian citizens and (2) inspect the relationship of SWB with MHL.

## 2. Materials and methods

### 2.1. Study design and sampling

In this analytical cross-sectional study, we recruited participants from April to December 2019 from different counties of Iran including large cities, small towns, and some villages. All adult users of online social media across the country could be included. Living in Iran, being literate, and having elementary internet skills were necessary for inclusion; a properly completed online form was considered proof that the inclusion criteria of being literate and able to use the internet are met. No exclusion criteria were considered. We used a convenient sampling method to collect data by sharing the study materials online. A sample size of 1,500 was calculated using the formula of estimating a mean in which n is the sample size, *α* is the expected type I error, *σ* is the standard deviation, and *d* is the marginal error. In this study *α* was considered 0.05, *σ* was estimated 5 according to previous studies in Iranian populations, and d was considered 1 (25, 26).


$$n=\frac{{\left(Z_{1-\frac{\alpha }{2}}\right)}^{2}\times \sigma^{2}}{d^{2}}$$

### 2.2. Assessment

An online form containing required informed consent, baseline characteristic questions, and three questionnaires were prepared. We used two instruments to evaluate MHL; one for evaluating positive MHL and the other for measuring other aspects of MHL. The 3rd questionnaire was a measure of SWB. The baseline questions inquired about the history of mental health service use and the individual’s perceived mental health status. Socioeconomic status was estimated by calculating the Household Crowding Index by dividing the number of the individual’s home mates by the number of rooms in the home (27). The time needed to complete the form was about 20 min. The explanation and instruction were summarized in a text message and disseminated through online social networks like WhatsApp and Telegram messengers.

### 2.3. Measurements

#### 2.3.1. WHO-Five well-being index (WHO-5)

This short and practical instrument is extracted from the longer 28 and then 10-item questionnaires (28). It has five non-invasive six-point agreement scale questions with scores from 0 to 5. These scores are summed up to result in the WHO-5 score, which is recommended to multiply by 4 to shape into a 0–100 score range. A higher score indicates better subjective well-being (SWB) in the past 2 weeks, and less than half of the total score shows poor well-being. WHO-5 has shown high clinometric validity and is sensitive to interventions. It is considered a useful final outcome scale of different well-being perspectives, i.e., hedonic and eudaimonic. WHO-5 is translated into more than 30 languages and is used widely across different cultures in the world. Although it was not developed for clinical applications, studies showed that WHO-5 scores of ≤ 50 are a good screening tool for clinical depression, with sensitivity and specificity of 0.86 and 0.81, respectively (28). The Persian version of WHO-5 is valid and reliable, with Cronbach’s alfa of 0.89 among university students and 0.91 among psychiatric outpatients (25, 26).

#### 2.3.2. Mental health literacy scale (MHLS)

Mental health literacy scale is one of the recommended instruments to evaluate MHL, which covers its various aspects except for positive MHL (29, 30). It has 35 questions with four or 5-point Likert scales and is shown to be valid and reliable with Cronbach’s alfa of 0.87. The number of questions in the validation and adaptation process of the Persian version is reduced to 23, resulting in a score range of 23–106. This Persian version is also reported to be valid and reliable (31).

#### 2.3.3. Mental health positive knowledge (MHPK)

To have a complete assessment of MHL, we also used MHPK, a measure of positive MHL. It is tested in Norwegian youth and showed good validity and reliability with McDonald’s omega of 0.84. It has ten items with a six-point agreement scale from 0 to 5, and their mean makes the total positive MHL score. Higher scores show better awareness about mental health promotive factors. The Persian version of MHPK has demonstrated good validity and reliability with Cronbach’s alfa of 0.81 (32).

### 2.4. Statistical methods

The main variables are described using mean, standard deviation (SD), and 95% confidence interval (95% CI), and frequency percentages are reported for categorical variables. Pearson correlation test is used to investigate the potential relationship between SWB and MHL scores and other quantitative measures. The association of SWB to other variables was tested using the independent-samples t-test. A multiple linear regression model was recruited to explore the amount of variance of SWB accounted for by MHL measures and two other mental health-related variables assessed in this study controlling for their interactions. SPSS Statistics for Windows, version 16 (SPSS Inc., Chicago, IL, United States) is used for these analyses, and the level of statistical significance and statistical power is considered to be 0.05 and 80%, respectively.

### 2.5. Ethics statement

The principles of the Helsinki Declaration were observed in this study. Confirming the informed consent part of the online form was required before completing it. An e-mail address was provided in the online form to answer any questions about confidentiality or problems in working with the form. Participants’ data were anonymous and protected confidentially. The research protocol was approved with the ethics reference number of IR.IUMS.FMD.REC.1397.173 by the medical ethics committee of Iran University of Medical Sciences.

## 3. Results

A total of 1,682 individuals participated in the study with a mean age of 25.99 (ranging from 18 to 69, SD 9.14). Most of the participants were female and 467 (28.1%) were male. The mean Household Crowding Index (HCI) was 1.12 (SD 0.57). Table 1 summarizes other baseline characteristics and associated WHO-5 scores and their association’s significance level.

**Table 1:** Baseline characteristics and associated WHO-5 scores; statistically significant differences are marked by the * sign.

			WHO-5	
		Frequency (Valid %)	Mean (SD)	*p*-Value
Gender	Females	1,196 (71.9%)	49.91 (20.67)	0.493
	Males	467 (28.1)	50.69 (21.44)	
Marriage	Single	927 (60.3%)	49.06 (20.97)	0.022*
	In a relationship	202 (13.1%)	52.49 (20.87)	
	Married	374 (24.3%)	50.77 (19.70)	
	Divorced	35 (2.3%)	42.62 (18.12)	
University education	No	358 (21.5%)	50.84 (22.35)	0.485
	Yes	1,309 (78.5%)	49.96 (20.51)	
Mental illness in acquaintances	No	885 (57.4%)	51.82 (20.52)	<0.001*
	Yes	656 (42.6%)	47.22 (20.53)	
Perceived mental health	Completely Ill	33 (2.1%)	33.33 (22.84)	<0.001*
	Partially Ill	262 (16.9%)	33.53 (17.53)	
	Partially Healthy	931 (60.2%)	50.05 (18.50)	
	Completely Healthy	320 (20.7%)	64.30 (17.56)	
Mental health service use	No	764 (49.5%)	51.69 (21.03)	0.001*
	Yes	780 (50.5%)	48.09 (20.17)	

The mean WHO-5 score was 50.19 (SD 20.92) with 95%CI of 49.18–51.19 and a range of 0 to 100. The number of participants with a WHO-5 score below 50—a known depression screening cut-off—was 839 (50.4%).

As shown in Table 1, SWB showed a significant positive association with higher perceived mental health status. The history of mental health service use and familiarity with someone suffering from mental illness was associated with lower SWB; the differences, however, were small. Individuals who described their marital status as “in a relationship” showed significantly higher SWB than divorced participants. Age or HCI were not significantly correlated to SWB. SWB was also not different among genders or education groups.

The mean MHLS score was 71.37 (SD 8.33) with 95%CI of 71.00–71.89. The mean MHPK score was 4.21 (SD 0.72) with 95%CI of 4.20–4.28. Pearson’s test showed a significant but very small correlation between WHO-5 and both MHL measures; it was correlated to MHLS with *r* = 0.062 (*p* = 0.021) and to MHPK with *r* = 0.073 (*p* = 0.003).

Using a multiple regression model, we tested the amount of variance of WHO-5 predictable by including these four independent variables: MHLS, MHPK, previous mental health service use, and mental illness in acquaintances. These factors are somehow related to mental health system services. Perceived mental health was not included as it is another subjective measure of one’s well-being. Although the model and all four variables were statistically significant, the resulting *R* square was 0.03.

## 4. Discussion

In this study, we estimated subjective well-being (SWB) in a sample of Iranian citizens. Furthermore, we explored the relationship of SWB with mental health literacy (MHL). The average SWB score was around half of the total possible score. More than half of the participants’ SWB was below the screening cut-off for clinical depression (28). Although statistically significant, the correlation of SWB with both MHL measures was small.

The level of SWB revealed in this study is low. Except for an investigation of mentally ill people, all previous studies that used WHO-5 in Iranian populations reported higher scores (26). In an analysis of people with infertility in 2019, 44.3% scored below the instrument’s cut-off, and in a sample of medical students studied in 2017, the below cut-off frequency was 34%; this number in our study was 50.4% (33, 34). On the other hand, in a nationwide survey in 2017 using Oxford Happiness Questionnaire, Montazeri, et al. reported an average score of 4.08 out of 6, labeled as “rather happy, pretty happy,” in 14,292 Iranian young adults. We expected better SWB results in our investigation because similar to the 2017 survey, participants in the current study were a sample of the general population, not ill or at-risk individuals. Our results, however, are similar to other recent studies that reported Iranian citizens’ happiness as relatively low and the recent World Happiness Report (11, 12, 14).

The lower SWB of divorced individuals in the current study is compatible with previous results in Iranian populations (12). However, a previous systematic review of the literature has shown the complicated relationship between SWB and divorce (35). Future investigations among Iranian divorced couples are needed to understand this less studied field. This study’s lack of difference in SWB scores among genders and education levels is consistent with current well-being knowledge and with previous investigations in Iranian populations (4, 12, 15).

Socioeconomic status is a determinant of SWB, so we used the Household Crowding Index (HCI) to estimate it (4, 12, 15). No significant association, however, was found between SWB and HCI in our study. This may show that our sampling has been limited to one social class, especially regarding the high education level of respondents, or may raise some questions about using HCI in Iranian populations despite satisfactory results in other societies (27).

A limited number of studies have inspected the relationship between MHL and SWB. Gorczynski et al. explored the association of MHLS and the Warwick–Edinburgh Mental Well-Being Scale in the United Kingdom in two populations, including university students and sports coaches, and found no correlation (8, 20). A similar result was found in an investigation of Australian adolescents using MHLS and EPOCH Measure of Adolescent Well-Being (9). We found a small significant correlation between MHLS and WHO-5 which may be explained by our different SWB instruments or by the much smaller sample size of those studies. We also detected a small significant correlation between positive MHL and SWB. This was similar to the findings of Bjørnsen et al.; they examined the association of positive MHL with another measure of well-being in 1888 Norwegian adolescents and found a small significant correlation (*r* = 0.17, *p* < 0.01).

As it is evident that MHL can be improved through education, it may be tempting to consider it a practical solution to enhance the SWB of individuals and communities (18, 24). However, the limited studies mentioned above do not support such a hypothesis. Although Bjørnsen et al. (21) concluded that positive MHL education could improve youth’s well-being, small correlation coefficients in Norwegian and Iranian samples indicate many other influential contributing factors. Our multiple regression model showed that all four mental health-related variables assessed in this study—including MHL measures, history of mental health service use, and relationship with a mentally ill person—only account for 3% of the SWB variance. This finding is in line with the current science of well-being which has shown that satisfactory SWB is not merely the product of good mental health. In other words, SWB cannot be reduced to good mental health by neglecting its other strong contributors such as good physical health, employment and high income, successful social relationships, fruitful spiritual and religious tendencies, and personality and thinking patterns (4, 15). Therefore, although MHL education benefits individuals’ mental health, it would not be a solitary solution to improve SWB. To address the low well-being of Iranians, a comprehensive plan which addresses scientifically approved determinants of well-being as a system would be an immediate necessity (11, 12, 15).

This study, along with presenting a recent SWB estimation of 1,682 Iranians, was the first study investigating the relationship between MHL and SWB in Iran and was one of the few in the world. In this study, we used a relatively big sample size. However, the sampling method is non-random, and the frequency of female and educated participants is higher than the general population. Therefore, the findings may only be generalized cautiously to educated citizens. Using a brief measure of well-being that cannot discriminate between hedonic and eudaimonic domains of SWB is another limitation of this study. Despite using this short instrument, overall, the participants needed 20 min to complete the form. This can be a source of bias as individuals with more interest or higher SWB may be selected. However, using the online form allowed participants to fill it in at the time and place they preferred and this may reduce the mentioned problem. Finally, although we inspected some of the external determinants of SWB, we did not explore its mental or cultural determinants.

This study indicates that the subjective well-being of half of the educated Iranian citizens in this sample is poor; this population is screen-positive for clinical depression and needs further mental health evaluations. The high frequency of poor well-being in this educationally successful population may warn of a national mental health and well-being problem that requires comprehensive assessments and interventions. Quite a small correlation between well-being and MHL measures confirms that well-being-improving programs cannot be reduced to mental health education interventions. A multicomponent systemic approach to improve scientifically approved determinants of well-being would be necessary.

## Data availability statement

The raw data supporting the conclusions of this article will be made available by the authors, without undue reservation.

## Ethics statement

The studies involving human participants were reviewed and approved by the medical ethics committee of Iran University of Medical Sciences. The patients/participants provided their written informed consent to participate in this study.

## Author contributions

SM contributed to the design of the work, data acquisition, analysis, interpretation, and drafting of the manuscript. MR and MA-A contributed to the conception and design, data interpretation, and revising of the manuscript critically. EK, ZJ, and SE contributed to designing the study, data collection, statistical analysis, and constructing the final manuscript. All authors contributed to the article and approved the submitted version.

## Conflict of interest

The authors declare that the research was conducted in the absence of any commercial or financial relationships that could be construed as a potential conflict of interest.

## Publisher’s note

All claims expressed in this article are solely those of the authors and do not necessarily represent those of their affiliated organizations, or those of the publisher, the editors and the reviewers. Any product that may be evaluated in this article, or claim that may be made by its manufacturer, is not guaranteed or endorsed by the publisher.
